# Effects of Co-Processing Sewage Sludge in the Cement Kiln on PAHs, Heavy Metals Emissions and the Surrounding Environment

**DOI:** 10.3390/ijerph15040698

**Published:** 2018-04-08

**Authors:** Dong Lv, Tianle Zhu, Runwei Liu, Xinghua Li, Yuan Zhao, Ye Sun, Hongmei Wang, Fan Zhang, Qinglin Zhao

**Affiliations:** 1 School of Space and Environment, Beihang University, Beijing 100191, China; lvdong427@126.com (D.L.); lingxinghua@buaa.edu.cn (X.L.); zhaoyuan01227@sina.com (Y.Z.); suny@buaa.edu.cn (Y.S.); 2 Beijing Enterprises Holding Environment Technology Co., Ltd., Beijing 100124, China; ws050521@126.com; 3 Chinese Research Academy of Environmental Sciences, Beijing 100012, China; wanghm5188@sina.com (H.W.); zhangfan5188@vip.sina.com (F.Z.); 4 School of Materials Science and Engineering, Wuhan University of Technology, Wuhan 430070, China; zhaoqinglin@whut.edu.cn

**Keywords:** cement plant, co-processing sewage sludge, PAHs, heavy metals, emission, surrounding environment

## Abstract

To understand the effects of co-processing sewage sludge in the cement kiln on non-criterion pollutants emissions and its surrounding environment, the flue gas from a cement kiln stack, ambient air and soil from the background/downwind sites were collected in the cement plant. Polycyclic aromatic hydrocarbons (PAHs) and heavy metals of the samples were analyzed. The results show that PAHs in flue gas mainly exist in the gas phase and the low molecular weight PAHs are the predominant congener. The co-processing sewage sludge results in the increase in PAHs and heavy metals emissions, especially high molecular weight PAHs and low-volatile heavy metals such as Cd and Pb in the particle phase, while it does not change their compositions and distribution patterns significantly. The concentrations and their distributions of the PAHs and heavy metals between the emissions and ambient air have a positive correlation and the co-processing sewage sludge results in the increase of PAHs and heavy metals concentrations in the ambient air. The PAHs concentration level and their distribution in soil are proportional to those in the particle phase of flue gas, and the co-processing sewage sludge can accelerate the accumulation of the PAHs and heavy metals in the surrounding soil, especially high/middle molecular weight PAHs and low-volatile heavy metals.

## 1. Introduction 

Rapid urbanization has increased the formation of municipal sewage sludge in China in recent years [1,2]. Given the sewage sludge contains nitrogen-containing compounds, organic matters and heavy metals [3], proper disposal should be employed to avoid environmental problems. Coincidentally, both a high temperature and alkaline environment are provided in the cement kiln so that most of the pollutants in the sewage sludge can be effectively destroyed or solidified to reduce the harm to the environment [4,5,6]. On the other hand, sewage sludge can be taken as an alternative fuel and raw material because it has a certain calorific value and contains SiO_2_, CaO, Al_2_O_3_, Fe_3_O_4_ and other cement components [7]. Therefore, the co-processing of sewage sludge in the cement kiln, as an environmentally friendly option [3], has been widely practiced in a number of developed countries and has also attracted increasing attention in China [8]. 

Various air pollutants, including particles, SO_2_, NO*_x_*, as well as non-criterion pollutants such as heavy metals (HMs) or semi-volatile organic compounds, are formed during cement production [7,9]. In recent years, heavy metals and semi-volatile organic compounds have become the focus of more and more concern because of their notable toxicity [10]. Some investigations have indicated that the composition of dioxins in flue gas is almost unchangeable, but its emission amount slightly increases due to co-processing the sewage sludge [5,6,11]. For heavy metals, the removal efficiency of non-volatile heavy metals was almost 100% when sewage sludge was co-processed in cement kiln, but the volatile heavy metals, especially Hg, cannot be removed completely by the process [6,12]. In addition, polycyclic aromatic hydrocarbons (PAHs) emissions also show a trend of increase [5,12,13,14].

Heavy metals and semi-volatile organic compounds emitted from the cement plant into the atmosphere can be transmitted to the human body directly through inhalation. Moreover, they can also cross environmental-media boundaries, becoming distributed in soils, vegetation, etc. [15]. As a result, human health can be indirectly harmed through different pathways. High levels of PAHs were found in the air around a cement plant in which kilns were operated [16]. Some studies also highlighted the negative impact of cement production on soil and vegetation growth [17]. Because of the difference of raw materials, fuels, composition of sewage sludge and even management level, some studies argued that the impact changes on the environmental and local population were not observed after co-processing sewage sludge in cement kiln [3].

To further understand the effects of co-processing sewage sludge in the cement kiln on PAHs and heavy metals emissions and its surroundings, a series of samples from flue gas of cement kiln and the surrounding air/soil were collected, PAHs (the 16 priority PAHs stipulated by US-EPA) and heavy metals [including arsenic (As), cadmium (Cd), cobalt (Co), chromium (Cr), copper (Cu), manganese (Mn), nickel (Ni), lead (Pb), tin (Sn), antimony (Sb), vanadium (V), and zinc (Zn)] of these samples were analyzed. Meanwhile, the relationships between PAHs/heavy metals emissions and their concentrations in air/soil were explored in this paper.

## 2. Materials and Methods

### 2.1. A Brief Introduction to the Cement Production Line

The cement plant investigated in this work, Taihang Qianjing, was built in the southern suburb of Beijing, China in January 2003 and equipped with a new suspension preheater (NSP) cement production line with a clinker production capacity of 3200 t·d^−1^. The production line is mainly composed of three parts: two groups of five-stage cyclone preheater system, a precalciner (836 °C–927 °C) and a rotary kiln (Φ4.3 m × 66 m). The production line is also equipped with selective non-catalytic reduction (SNCR) denitration system and bag filter. In the end of May 2015, the co-processing sewage sludge in cement kiln was carried out with a sewage sludge treatment capacity of 100 t·d^−1^. The PAHs and heavy metals in raw materials, fuels and sewage sludge were analyzed. The results are shown in Table 1.

### 2.2. Sampling

**Figure 1:**
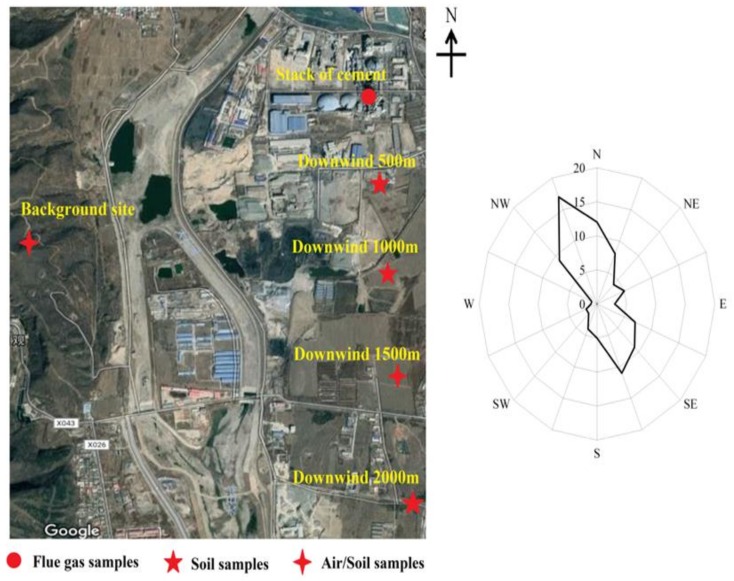
Sampling sites for flue gas and air/soil samples.

Sampling sites for flue gas and air/soil samples are shown in Figure 1. Flue gas samples were isokinetically collected from the stack of the cement kiln with a self-made sampling system [14] under conditions of being with and without co-processing sewage sludge, respectively. A tube-type quartz fiber filter (QFF), pretreated for 4 h by heating it at 450 °C in a muffle furnace, was used to collect particle matter, and a quartz cartridge packed with 20 g XAD-2 resin was used to adsorb the gaseous pollutants. Both cooling water and the residual in the inner wall of the sampling pipe containing gaseous pollutants were also collected by washing with n-hexane. 

Air samples were simultaneously collected at both the background site (1500 m to the west by south of the cement kiln stack) and downwind site (1500 m to south by east of the cement plant stack) with a low-volume air sampler (MiniVol, Airnetrics, Springfield, OR, USA). There was no other distinct air pollution source near this cement plant. During sampling, the air was drawn through a quartz fiber filter with a diameter of 47 mm to collect particle matter and then through polyurethane foam (PUF) to collect gaseous pollutants. Sampling duration and volume were 48 h and 14.5 m^3^ for each sample. Prior to sampling, QFFs were baked at 450 °C overnight and stored in aluminum foil packages until used. PUF plugs were cleaned by dichloromethane (DCM), dried in an oven at 50 °C, placed in glass jars and stored in a freezer until used without being exposed to ambient air.

After the above-mentioned sampling, the QFF filters were packed with aluminum foil and stored in a freezer (−4 °C). PUF plugs, quartz cartridges and washing solution (cooling water and residual in the inner wall of sampling pipe) were placed into containers and stored in a freezer (−18 °C).

Soil samples were collected in both the background site (1500 m to the west by south of the cement kiln stack) and downwind sites (500 m, 1000 m, 1500 m and 2000 m to south by east of the cement plant stack) in May 2015 (before co-processing sewage sludge) and May 2016 (after co-processing sewage sludge for one year), respectively. About 500 g of soil sample was obtained from a surface-to-5 cm depth at each sampling site. The soil samples were also immediately packed in a brown paper bag, then dried at room temperature, sieved with a 2-mm mesh sieve and stored until analysis. 

### 2.3. Analytical Procedure

For the particle matter from flue gas, air and soil, both PAHs and heavy metals were analyzed. For the gaseous pollutants from flue gas and air, only PAHs were analyzed because hardly any heavy metal considered in this investigation exists in the gas phase. The PAHs contained in QFF, quartz cartridges, PUF and washing solution were extracted with dichloromethane in an ultrasonic bath. The extraction was then concentrated with a rotary evaporator, purified by using solvent exchanged to *n*-hexane, reconcentrated with N_2_ and diluted to the certain volume with *n*-hexane. The 16 priority PAHs stipulated by US-EPA were determined by gas chromatographic mass spectrometry (GC-MS, Trace GC Polaris Q, Thermo Fisher, Waltham, MA, USA, with DB-5 ms, 30 m × 0.25 mm × 0.25 μm) in selected ion monitoring mode. PAHs can be classified into low molecular weight PAHs (LMW PAHs) with 2- and 3-rings, middle molecular weight PAHs (MMW PAHs) with 4-rings and high molecular weight PAHs (HMW PAHs) with 5- and 6-rings. 

For heavy metals analysis, half of QFF and 0.1 g of dried soil were digested in hermetic Teflon bombs with 8 mL mixture of HNO_3_ and HF with ratio of 5:3 and 2 mL HNO_3_, respectively. Then, they were treated in a microwave digestion system (Mars 6, CEM, Matthews, NC, USA) for 10 min until reaching 165 °C and were kept at this temperature for 20 min. After cooling, the extractions were filtered and made up to 10 mL and 25 mL with ultrapure water, respectively. The concentrations of As, Cd, Co, Cr, Cu, Hg, Mn, Ni, Pb, Sn, V and Zn were determined by inductively coupled plasma spectrometry (ICP-MS, Perkin Elmer Elan 6000, Waltham, MA, USA). Generally, the heavy metals can be classified into non-volatile heavy metals (NV-HMs) including As, Co, Cr, Cu, Mn, Ni, Sn, Sb, V and Zn, and low-volatile heavy metals (LV-HMs), including Cd, Pb in this investigation [18,19].

## 3. Results and Discussion

### 3.1. Effects of Co-Processing Sewage Sludge on Polycyclic Aromatic Hydrocarbons and Heavy Metals Emissions

In order to investigate the effects of co-processing sewage sludge on non-criterial pollutant emissions, the flue gas samples from the cement kiln stack were collected and analyzed in 0 and 100 t/d co-processing sewage sludge amount. Table 2 gives PAHs concentrations in the gas phase and particle phase and heavy metals concentrations in the particle phase. It can be seen that the co-processing sewage sludge results in an increase in PAHs and heavy metals emissions, especially MMW PAHs, HMW PAHs, Cd and Pb, whereas it does not change their compositions and distribution patterns in the flue gas significantly.

#### 3.1.1. Polycyclic Aromatic Hydrocarbons Emissions

**Figure 2:**
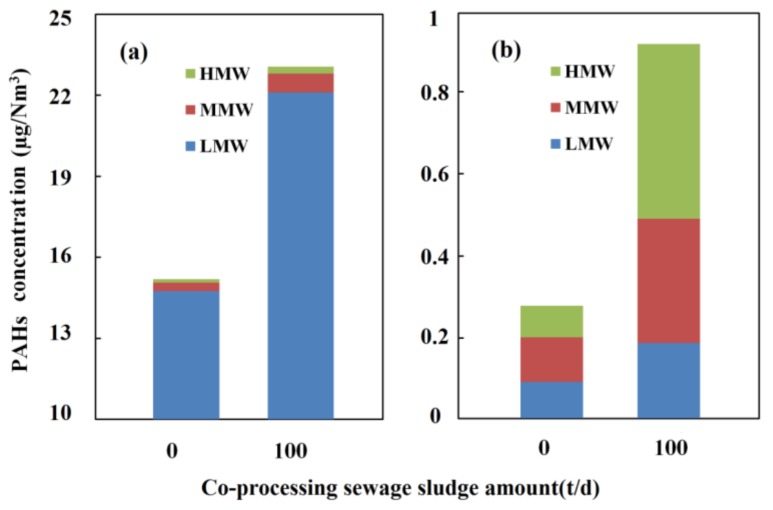
The low molecular weight (LMW) PAHs, middle molecular weight (MMW) PAHs and high molecular weight (HMW) PAHs distributions in gas phase (**a**) and particle phase (**b**) of the flue gas.

As shown in Table 2, the total PAHs concentrations of flue gas without and with co-processing sewage sludge are 15.472 μg/m^3^ and 23.981 μg/m^3^, respectively. To intuitively compare the PAHs distribution in the gas phase and particle phase, as well as the effects of co-processing sewage sludge, Figure 2 is given. It can be seen from Figure 2 and Table 2 that PAHs mainly exist in the gas phase, accounting for 98.2% and 96.1% without and with co-processing, respectively. In the gas phase, the LMW PAHs are the predominant congener, accounting for 96.9% and 95.8% without and with co-processing, respectively, which is consistent with the previous investigations and is attributed to the fact that MMW and HMW PAHs can be decomposed into LMW PAHs under the condition of the high temperature in cement precalciner, and that LMW PAHs prefer to exist in the gas phase [20]. Moreover, the co-processing sewage sludge results in a pronounced increase of total PAHs concentrations in particle phase, being up to 228.7%. In particle phase, HMW PAHs concentration increases by 455% because of co-processing sewage sludge, which may be attributed to the fact that the sewage sludge feeding into the cement kiln contains a certain amount of HMW PAHs (Galvez et al., 2007; Dai et al., 2014). On the other hand, the HMW PAHs are absorbed on the surface of particle more easily.

#### 3.1.2. Heavy Metals Emissions

As shown in Table 2, Cr is the most abundant element, followed by Pb, Cu, Mn, and so on without co-processing sewage sludge. The co-processing sewage sludge results in a significant increase of Cd and Pb emissions: up to 260.9% and 117.0%, respectively. However, no significant change occurs for other heavy metal emissions because of co-processing sewage sludge. So, Cd and Pb may be the fingerprints left by co-processing sewage sludge. As is well known, the behavior of heavy metals in the heating process largely depends on their volatility [9]. The low volatile compositions in the sewage sludge, such as Cd and Pb, may make a much larger contribution to an increase of heavy metal emissions because they easily evaporate and enter flue gas during heat decomposition and calcination while the non-volatile metals mainly remain in the cement clinker. The low volatile compositions become the submicron metal particles or are adsorbed on the surface of the fine particles which cannot be effectively captured by conventional dust removal equipment such as electrostatic precipitator and bag filter during cooling after emitting cement production system. 

According to the above-mentioned results and discussion, it can be seen that more attention should be paid to the HMW PAHs and low-volatile heavy metals in particle phase when monitoring the non-criterial air pollutant emissions from cement production with co-processing sewage sludge.

### 3.2. Effects of Co-Processing Sewage Sludge on Ambient Air

In order to investigate the effects of co-processing sewage sludge in the cement kiln on the ambient air, ambient air samples including both gas phase and particle phase were synchronously collected in two sampling sites, i.e., background and downwind sites as described in Section 2.2. Table 3 gives the concentrations of PAHs and heavy metals in the samples. It can be seen that the PAHs and heavy metals concentrations in the downwind site are higher than those of the background site, especially the HMW PAHs, Cd and Pb, whether sewage sludge is co-processed or not. Actually, the concentration difference between the background site and downwind site would become more obvious if it had not been for the wind direction oscillation during sampling. It is clear that the ambient air, to a certain extent, was affected by this cement plant. On the other hand, the co-processing sewage sludge results in the increase of PAHs and heavy metals concentrations in the ambient air, having a positive correlation between the emissions and ambient air.

#### 3.2.1. Polycyclic aromatic hydrocarbons Concentrations in Ambient Air

**Figure 3:**
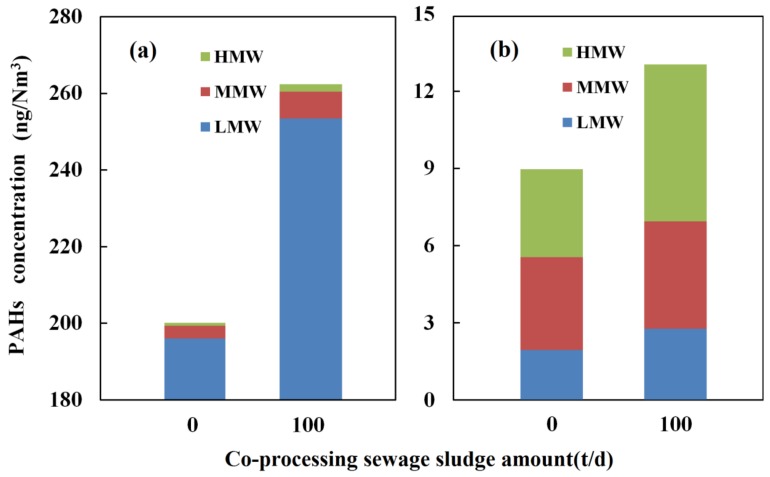
Polycyclic aromatic hydrocarbons concentration difference between downwind and background sites in gas phase samples (**a**) and particle phase samples (**b**) of ambient air.

To intuitively reflect the contribution of cement production and co-processing sewage sludge on PAHs concentration in the ambient air, Figure 3 gives the PAHs concentration differences between the downwind site and background in both the gas phase and particle phase. It can be seen that the PAHs increment in the gas phase is much larger than that in the particle phase, whether the sewage sludge is co-processed or not, and the LMW PAHs increment accounts for the overwhelming majority of the total PAHs increment in gas phase, which agrees with the previous researches [21]. Moreover, co-processing sewage sludge results in a significant increase of the PAHs increment, especially in the gas phase. However, the increments of MMW and HMW PAHs account for the majority of the total increment in particle phase, which is similar to the PAHs distribution in particle phase from the flue gas whether sewage sludge is co-processed or not, as shown in Figure 2. Clearly, the PAHs increment is caused by the PAHs emissions from the cement kiln because it is positively correlated with the increase of PAHs emissions from the cement production and co-processing sewage sludge.

#### 3.2.2. Heavy Metals Concentrations in Ambient Air

As shown in Table 3, the increments of Cd and Pb concentrations of downwind site relative to background site are 41.7% and 37.7% without co-processing sewage sludge and 92.8% and 55.0% with co-processing sewage sludge, respectively, being higher than those of other heavy metals, while Mn concentration is the highest, followed by Zn among the all heavy metals. Therefore, it can be concluded that the effects of Cd and Pb emissions from the cement production on ambient air is larger than those of other heavy metals whether the sewage sludge is co-processed or not. 

### 3.3. Effects of Co-Processing Sewage Sludge on Soil Environment

Soil is the ideal long-term monitor, and the accumulation in soil is the result of atmospheric dry or wet deposition, vapor diffusion and the loss of contaminants through various processes such as leaching or volatilization. In order to investigate the effects of co-processing sewage sludge on the soil environment, soil samples from the background site and four downwind sites were collected in this investigation in May 2015 (before co-processing sewage sludge) and May 2016 (after co-processing sewage sludge for one year), respectively. 

#### 3.3.1. Polycyclic Aromatic Hydrocarbons Concentrations in Soil 

**Figure 4:**
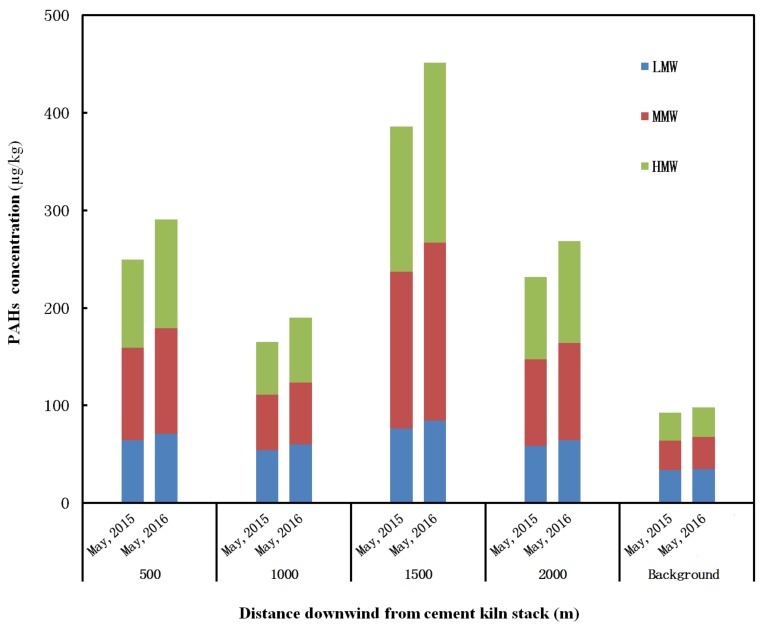
Polycyclic aromatic hydrocarbons concentration in soil in different sampling sites and periods.

Figure 4 illustrates the PAHs distributions in five sampling sites before co-processing sewage sludge (May 2015) and after co-processing sewage sludge for one year (May 2016). It can be seen that PAHs concentrations of downwind sites were significantly higher than that of the background site whether sewage sludge is co-processed or not, especially the MMW PAHs and HMW PAHs. By comparing the PAHs distributions in flue gas (Figure 2), it can be seen that the PAHs distribution with different molecular weight in the soil is similar to that in particle phase from flue gas (Figure 2b). Moreover, it can be also seen that the largest PAHs concentration in soil occurs in the sampling site of 1500 m downwind from the cement kiln stack. It, coincidentally, is the maximum ground-level PAHs concentration distance calculated by the atmospheric dispersion model, based on the flue gas parameters of the cement kiln and the local meteorological conditions near the cement plant. Clearly, the PAHs in the particle phase of flue gas may have an important influence on the PAHs concentration level in soil near the cement plant whether sewage sludge is co-processed or not.

In order to analyze the effects of co-processing sewage sludge on the soil environment, the annual accumulation amount of pollutants in the soil in the downwind site was evaluated as follows:

*M*_ave_ = (*C*_d(2015)_−*C*_b(2015)_)/12 (1)

*M*_2016_ = (*C*_d(2016)_−*C*_b(2016)_)−(*C*_d(2015)_−*C*_b(2015)_) (2)

where *C*_d(2015)_ and *C*_d(2016)_ are the average concentration of PAHs in the downwind site in May 2015 and May 2016, respectively; *C*_b(2015)_ and *C*_b(2016)_ are the average concentration of pollutants in the background site in May 2015 and May 2016, respectively. The results show that the annual average accumulation amounts of LMW PAHs, MMW PAHs and HMW PAHs in downwind sites between May 2015 and May 2016 (5.5 μg·kg^−1^·yr^−1^, 14.9 μg·kg^−1^·yr^−1^, 18.1 μg·kg^−1^·yr^−1^, respectively) are much larger than those before May 2015 (2.5 μg·kg^−1^·yr^−1^, 5.8 μg·kg^−1^·yr^−1^, 5.3 μg·kg^−1^·yr^−1^, respectively), which, to a certain extent, indicates that the co-processing sewage sludge in cement kiln can accelerate the PAHs accumulation in the surrounding soil because it increases the PAHs emissions. 

#### 3.3.2. Heavy Metals Concentrations in Soil 

**Figure 5:**
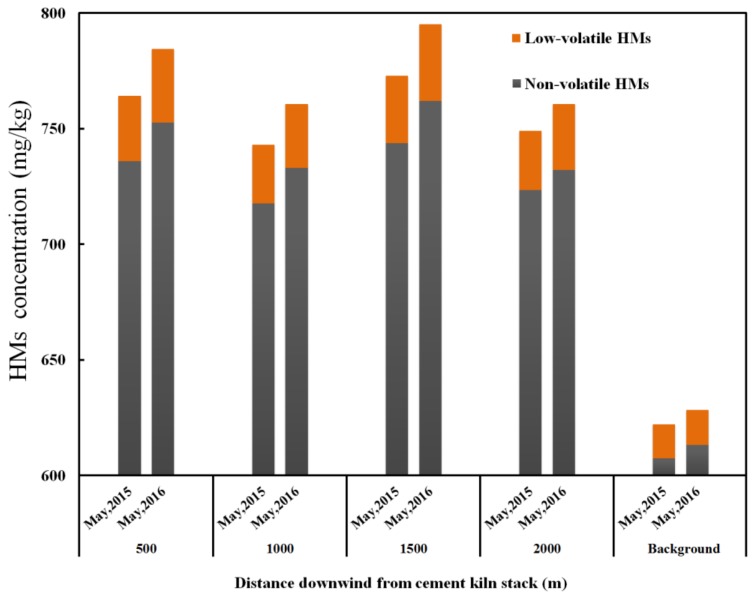
Heavy metals concentration in soil in different sampling sites and periods.

The analysis shows that the heavy metal with the largest concentration is Mn, followed by Zn, Cr, Pb and Cu, and that Sb was not detected in the soil samples collected in both May 2015 and May 2016. To clearly show accumulation of heavy metals in soil, Figure 5 gives the heavy metals distributions in five sampling sites before co-processing sewage sludge (May 2015) and after co-processing sewage sludge for one year (May 2016). Similar to the PAHs, the heavy metals concentrations in the downwind sampling sites are higher than those in the background sites. 

The maximum ground-level concentration distance of heavy metals is the same as that of PAHs whether sewage sludge is co-processed or not. The annual accumulation amounts of heavy metals were also calculated similarly by Formulas (1) and (2). The annual average accumulation amount of low-volatile heavy metals in downwind sites between May 2015 and May 2016 is 2.6 mg·kg^−1^·yr^−1^, being 5.3 and 2.6 times higher than that in background sites between May 2015 and May 2016 and in downwind sites before May 2015, respectively, which means co-processing sewage sludge in the cement kiln can accelerate the accumulation of low-volatile heavy metals in the surrounding soil because it increases the emissions of the low-volatile heavy metals. On the other hand, the average accumulation amount of non-volatile heavy metals between May 2015 and May 2016 is 10.8 mg·kg^−1^·yr^−1^, being similar to that before May 2015 (10.2 mg·kg^−1^·yr^−1^) in downwind soil sampling sites, which can be contributed to the decrease of non-volatile heavy metals loss by volatilization. In summary, the accumulation of heavy metals in the soil of the downwind near cement plant is notable. Thus, cement production may bring negative impacts on the surrounding soil environment, and the negative impacts become more serious because of co-processing sewage sludge.

## 4. Conclusions

Above 95% of all PAHs from the cement production flue gas exists in the gas phase, and the majority of these are LMW PAHs whether the sewage sludge is co-processed or not, which is attributed to the decomposition of MMW and HMW PAHs into LMW PAHs under the condition of the high temperature in the cement precalciner. The co-processing sewage sludge results in a significant increase of PAHs and heavy metals emissions. In particular, the emissions of HMW PAHs and low-volatile heavy metals such as Cd and Pb in the particle phase increase above twofold. Thus, the non-criterial pollutants emissions from cement production with co-processing sewage sludge should be paid more attention.

The PAHs and heavy metals emissions from the cement kiln have a positive correlation with their concentrations in the surrounding air/soil. The PAHs and heavy metals concentrations of ambient air or soil samples downwind are higher than those in the background. In the air samples, the components with a notable increase are HMW PAHs and low-volatile heavy metals in particle phase especially for co-processing sewage sludge. The annual accumulation amounts of PAHs and heavy metals in soil after co-processing sewage sludge are larger than those before co-processing sewage sludge, especially the HMW PAHs and low-volatile heavy metals. 

## Figures and Tables

**Table 1:** Polycyclic aromatic hydrocarbons and heavy metals concentrations in raw materials, fuels and sewage sludge.

	Cement Raw Materials (mg/kg)	Coal (mg/kg)	Sewage Sludge (mg/kg)
Nap	0.02	0.01	0.46
AcPy	0.01	0.03	0.05
Acp	0.01	0.02	0.06
Flu	0.02	0.06	0.20
PA	0.43	1.09	2.04
Ant	0.21	0.34	1.50
FL	0.34	0.55	1.13
Pyr	0.33	0.53	0.91
BaA	0.24	0.49	0.89
CHR	0.10	0.41	0.59
BbF	0.34	0.52	1.39
BkF	0.05	0.13	0.83
BaP	0.12	0.21	0.43
IND	0.02	0.03	1.90
DBA	0.64	1.11	2.12
BghiP	0.54	1.16	4.91
As	8.4	7.0	24.8
Co	0.2	0.7	1.6
Cr	12.5	27.3	178.2
Cu	217	262.1	318.1
Mn	474.2	632.1	828.9
Ni	14.1	19.3	35.8
Sb	1.0	2.7	4.2
Sn	0.1	0.2	0.5
V	45.2	30.4	21.3
Zn	46.8	83.2	172.3
Pb	9.3	16.5	83.4
Cd	1.2	2.4	13.4

**Table 2:** Polycyclic aromatic hydrocarbons and heavy metals concentrations (μg/Nm^3^) in the flue gas.

	Sewage Sludge 0 t/d	Sewage Sludge 100 t/d	Increment, %
Nap	9.721	13.683	40.8
AcPy	1.120	1.503	34.1
Acp	0.486	0.762	56.9
Flu	0.168	0.441	162.5
PA	3.271	5.433	66.1
Ant	0.200	0.472	136.5
LMW	14.818	22.294	49.1
FL	0.096	0.312	225.4
Pyr	0.149	0.186	24.3
BaA	0.105	0.281	168.8
CHR	0.101	0.228	126.5
MMW	0.450	1.006	123.4
BbF	0.042	0.151	260.3
BkF	0.013	0.063	395.1
BaP	0.037	0.119	220.7
IND	0.045	0.066	49.0
DBA	0.027	0.124	369.7
BghiP	0.041	0.157	260.3
HMW	0.204	0.680	234.2
∑PAHs	15.472	23.981	53.7
As	9.361	12.538	34.0
Co	0.972	1.258	29.4
Cr	65.315	69.289	6.1
Cu	36.166	38.576	6.7
Mn	27.251	28.471	4.5
Ni	9.734	13.047	34.0
Sb	3.956	4.029	1.9
Sn	2.395	2.597	8.4
V	4.170	4.772	14.5
Zn	24.046	26.928	12.0
NV-HMs	183.366	201.505	9.9
Pb	52.048	112.927	117.0
Cd	0.572	2.064	260.9
LV-HMs	52.620	114.991	118.5
∑HMs	235.986	316.496	34.1

**Table 3:** Polycyclic aromatic hydrocarbons and heavy metals concentrations in air samples (ng/Nm^3^).

	Sewage Sludge 0 t/d			Sewage Sludge 100 t/d		
	Background	Downwind	Increment, %	Background	Downwind	Increment, %
Nap	1052.6	1231.5	17.0	1089.9	1312.5	20.4
AcPy	12.7	15.4	21.5	14.5	18.7	29.4
Acp	16.3	19.7	21.1	17.5	23.8	36.3
Flu	20.0	23.0	15.3	24.3	30.8	26.7
PA	21.0	28.3	34.9	26.9	37.5	39.1
Ant	6.6	9.1	37.6	10.1	16.0	58.8
LMW	1129.1	1327.1	17.5	1183.2	1439.4	21.7
FL	3.6	4.7	29.4	4.3	6.3	46.4
Pyr	5.5	6.3	14.7	5.5	7.8	40.5
BaA	7.0	10.3	47.1	7.4	12.1	62.7
CHR	2.6	4.3	67.2	2.8	5.0	81.8
MMW	18.7	25.7	36.9	20.0	31.1	55.7
BbF	0.8	1.3	52.0	0.7	1.7	128.5
BkF	1.3	1.8	39.4	1.6	2.4	49.1
BaP	1.2	1.9	60.3	1.4	2.7	91.1
IND	1.3	1.8	45.1	1.4	2.5	73.0
DBA	1.4	2.2	53.7	1.8	3.2	81.8
BghiP	2.0	3.2	57.9	2.6	5.1	100.6
HMW	8.1	12.3	51.9	9.5	17.5	85.2
∑PAHs	1155.9	1365.0	18.1	1212.6	1488.0	22.7
As	9.8	11.7	19.6	11.2	13.6	21.1
Co	1.7	1.9	14.1	1.8	2.1	17.4
Cr	42.6	50.3	18.0	45.5	53.9	18.6
Cu	16.5	17.7	7.4	20.1	21.7	7.8
Mn	103.6	122.8	18.5	108.0	129.5	20.0
Ni	13.9	14.7	6.0	13.7	15.0	9.2
Sb	3.9	4.4	13.1	4.0	4.9	21.9
Sn	8.6	9.2	7.9	9.0	9.5	5.7
V	8.7	9.7	12.4	8.9	10.5	17.9
Zn	101.9	113.9	11.8	103.4	117.6	13.7
NV-HMs	311.0	356.4	14.6	325.6	378.2	16.1
Pb	32.9	45.3	37.6	39.5	61.2	55.0
Cd	0.7	1.0	41.7	0.9	1.8	92.8
LV-HMs	33.6	46.3	37.7	40.4	63.0	55.8
∑HMs	344.6	402.6	16.8	366.0	441.1	20.5
